# Enhancing armeniaspirols production through multi-level engineering of a native *Streptomyces* producer

**DOI:** 10.1186/s12934-023-02092-4

**Published:** 2023-04-28

**Authors:** Elena Heng, Yi Wee Lim, Chung Yan Leong, Veronica W. P. Ng, Siew Bee Ng, Yee Hwee Lim, Fong Tian Wong

**Affiliations:** 1 Molecular Engineering Laboratory, Institute of Molecular and Cell Biology (IMCB), Agency for Science, Technology and Research (A*STAR), 61 Biopolis Drive, #07-06, Proteos, Singapore, 138673 Singapore; 2 Chemical Biotechnology and Biocatalysis, Institute of Sustainability for Chemicals, Energy and Environment (ISCE2), Agency for Science, Technology and Research (A*STAR), 8 Biomedical Grove, Neuros, #07-01, Singapore, 138665 Singapore; 3 Singapore Institute of Food and Biotechnology Innovation (SIFBI), Agency for Science, Technology and Research (A*STAR), 31 Biopolis Way, Level 2, Nanos, Singapore, 138669 Singapore

**Keywords:** Natural products, Biosynthetic engineering, Upregulation, Armeniaspirols, *Streptomyces*, Fatty acyl-CoA synthase

## Abstract

**Background:**

Nature has provided unique molecular scaffolds for applications including therapeutics, agriculture, and food. Due to differences in ecological environments and laboratory conditions, engineering is often necessary to uncover and utilize the chemical diversity. Although we can efficiently activate and mine these often complex 3D molecules, sufficient production of target molecules for further engineering and application remain a considerable bottleneck. An example of these bioactive scaffolds is armeniaspirols, which are potent polyketide antibiotics against gram-positive pathogens and multi-resistance gram-negative *Helicobacter pylori*. Here, we examine the upregulation of armeniaspirols in an alternative *Streptomyces* producer, *Streptomyces* sp. A793.

**Results:**

Through an incidental observation of enhanced yields with the removal of a competing polyketide cluster, we observed seven-fold improvement in armeniaspirol production. To further investigate the improvement of armeniaspirol production, we examine upregulation of armeniaspirols through engineering of biosynthetic pathways and primary metabolism; including perturbation of genes in biosynthetic gene clusters and regulation of triacylglycerols pool.

**Conclusion:**

With either overexpression of extender unit pathway or late-stage *N*-methylation, or the deletion of a competing polyketide cluster, we can achieve seven-fold to forty nine-fold upregulation of armeniaspirol production. The most significant upregulation was achieved by expression of heterologous fatty acyl-CoA synthase, where we observed not only a ninety seven-fold increase in production yields compared to wild type, but also an increase in the diversity of observed armeniaspirol intermediates and analogs.

**Supplementary Information:**

The online version contains supplementary material available at 10.1186/s12934-023-02092-4.

## Background

Natural products (NPs) are highly bioactive molecules with widespread utility in therapeutic applications; for example, > 50% of current small molecule drugs are NPs or NP-derived [3, 11]. Recent genomics and bioinformatics analyses of Nature’s materials highlighted significant untapped potential in NP chemical diversity as the 400 K NPs characterized to-date represent only about 20% of Nature’s possible repertoire [15]. NP biosynthesis is highly regulated through nutritional, environmental signals, combined with pleiotropic and pathway-specific regulatory mechanisms. Thus, discovery and downstream applications of NPs are oftentimes hindered by their low production under laboratory conditions. To activate and regulate production of these rare NPs, our labs and others have developed various native strain and heterologous engineering strategies aided by synthetic biology techniques (i.e. CRISPR-Cas mediated editing, DNA capture and refactoring [8, 10, 20].

**Fig. 1:**
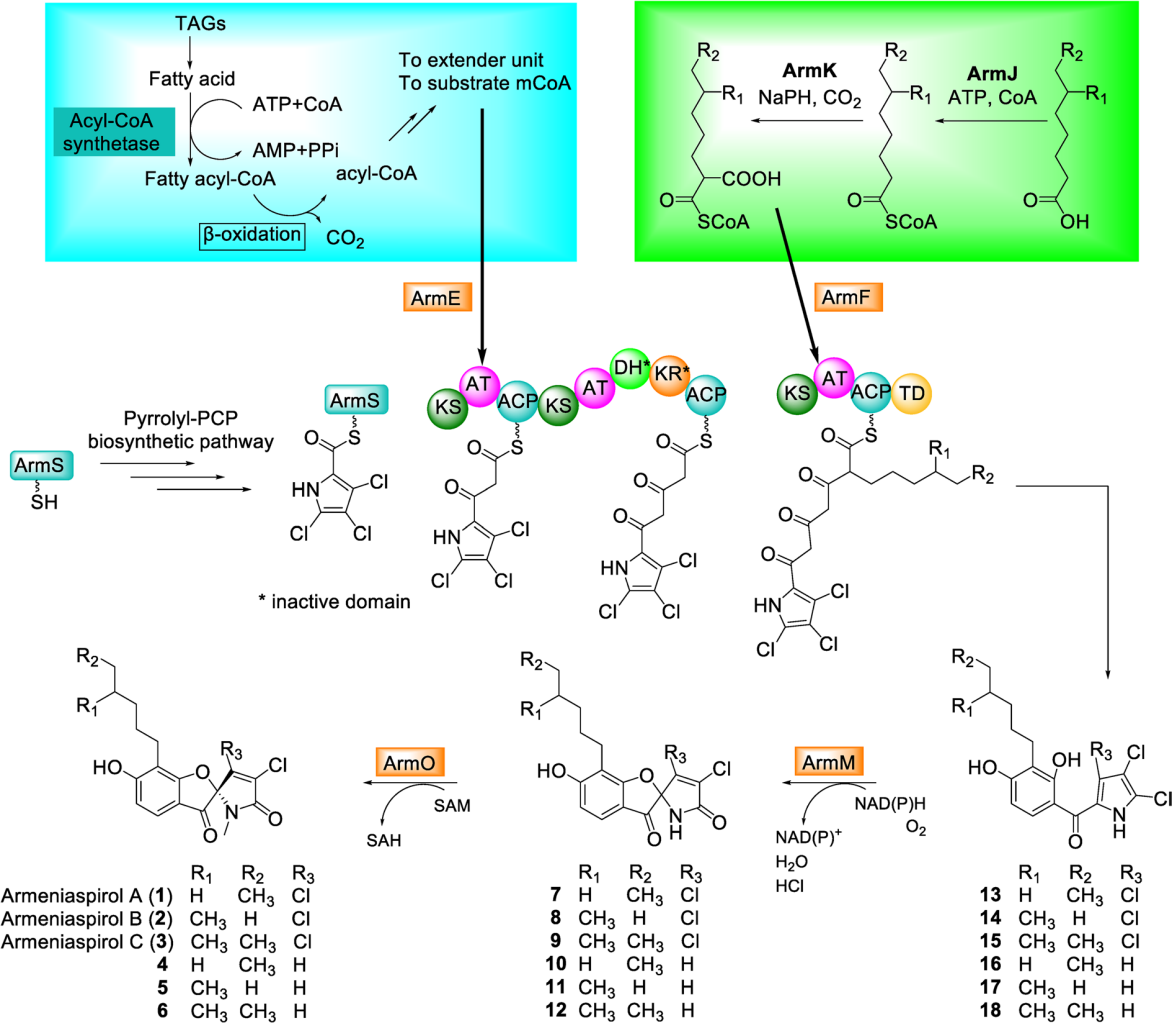
Biosynthetic pathway for armeniaspirols. Left inset: Fatty acid pathway [19]. Right inset: Putative 2-alkylmalonyl-CoA biosynthesis pathway

Armenispirols are a family of bioactive polyketide antibiotics with an unique chlorinated spiro[4.4]non-8-ene scaffold. Recent elucidation of the biosynthetic pathways for armeniaspirols in *Streptomyces armeniacus* have led to discovery and characterization of new biosynthetic enzymes and analogs [5, 12] for this family of NPs. Biosynthesis of armeniaspirols in *Streptomyces armeniacus* is initiated with the formation of a dichloropyrrolyl starter unit and a 2-alkylmalonyl CoA extender unit. Its biosynthetic gene cluster (BGC) includes three polyketide synthase modules, as well as a variety of tailoring enzymes responsible for chlorination, cyclization, and methylation, which together generate to the final chlorinated spiro[4.4]non-8-ene product (Fig. 1) [5, 12]. Novel analogs have also been generated through manipulation of the tailoring enzymes [12] and feeding of new acyl-CoA derivatives [21]. Besides anti-MRSA (Methicillin-resistant *Staphylococcus aureus*) and anti-VRE (vancomycin-resistant *Enterococcus*) bioactivity [2], its proposed mechanism of bacterial cell membrane disruption has also conferred armeniaspirol A potent bioactivity against multi-resistance gram-negative *Helicobacter pylori* [9].

Here, we examine strategies to enhance production of armeniaspirols in an alternative producer *Streptomyces* sp. A793 [7]. These include investigation of competing polyketide synthase, increasing precursors via primary metabolism or extender unit pathway and tailoring enzymes. Through engineering of the BGC and pleiotropic regulator overexpression, we demonstrate up to 97-fold enhancement of armeniaspirol production.

## Results

### Armeniaspirols in *Streptomyces* sp. A793

**Fig. 2:**
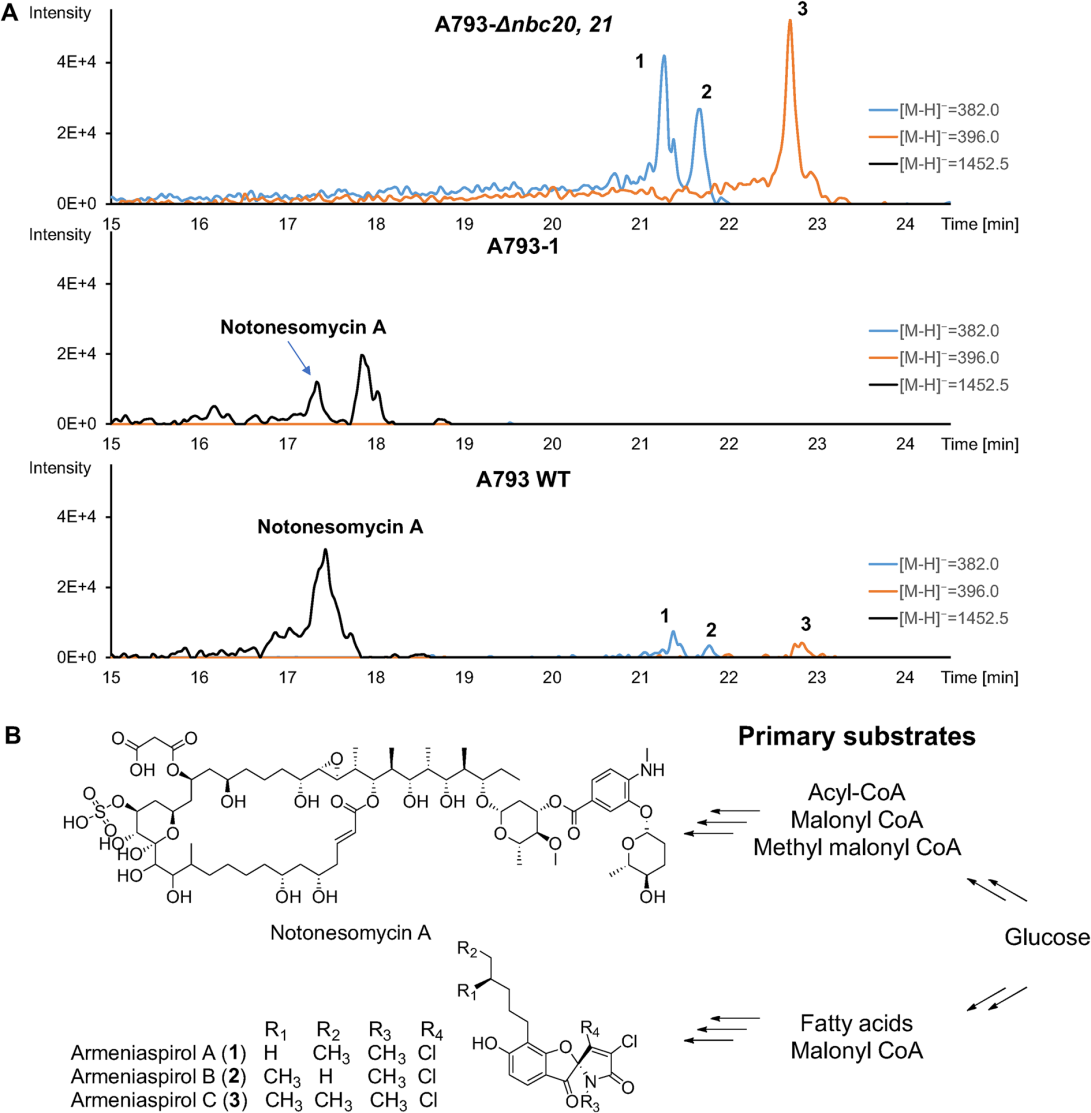
Armeniaspirols in *Streptomyces* sp. A793. **A** LCMS spectra comparison of notonesomycin BGC disrupted strain (A793-Δ*nbc20, 21*), polyketide module *armE* deleted strain (A793-1) and *Streptomyces* sp. A793 wild type strain (A793 WT). **B** Structures of notonesomycin and armeniaspirols with their respective substrates from the primary metabolism pathways

In a prior work examining notonesomycin production in *Streptomyces* sp. A793, we fermented a mutant strain (A793-Δ*nbc20, 21*, Additional file 1: Fig. S1), where the loading polyketide domains of notonesomycin BGC were deleted via CRISPR-Cas mediated editing [7]. In this mutant strain, the production of notonesomycin was disrupted but we also observed a sevenfold upregulation of armeniaspirols (**1**–**3**) production (Fig. 2A, Additional file 1: Fig. S1). In parallel, antiSMASH analysis of its genome also identified a putative gene cluster containing enzymes for biosynthesis of pyrrolomycin, 2-alkylmalonyl-CoA extender unit and polyketide synthases (Fig. 1, Additional file 1: Table S1) that corresponded to armeniaspirols producing BGCs (MiBiG IDs: BGC0001934, BGC0002022) as identified by two other separate groups [5, 12]. The native BGC was also verified by deletion of the polyketide module, *armE*, which removed armeniaspirol production (Fig. 2A, Additional file 1: Fig. S2, A793-1).

The observation of enhanced armeniaspirol production when notonesomycin BGC was disrupted led us to hypothesize that polyketide synthases within the gene clusters of notonesmycin and armeniaspirols might be competing for similar primary substrates, such as acyl-CoAs (Fig. 2B). With only seven-fold yield improvement compared to *Streptomyces* sp. A793 wild type strain, we reasoned that limits for armeniaspirol production have not been reached and it could be further enhanced.

### Strain engineering strategies

Armed with our initial hypothesis that precursors might be limiting, we investigated multiple approaches to improve precursor levels and examined their impacts on armeniaspirol production level (Fig. 1). A recent observation of intracellular triacylglycerols degradation for channelling carbon flux towards polyketide via key enzyme fatty acyl-CoA synthase (FAS, SCO6196, [19] led us to include FAS overexpression as a strategy to improve precursor levels. In this previous study by Wang et al. on *Streptomyces coelicolor* M145, the expression of SCO6196 resulted in an estimated two-fold to three-fold increase in carbon fluxes directed towards malonyl CoA and methylmalonyl CoA, as well as increased levels of reducing co-factors equivalents (NADH). Besides increasing precursors from primary metabolism, we also wondered if production of the unique extender, 2-alkylmalonyl CoA, could be limiting. Within the BGC, *armJKL* is highly similar for *revRST* which encodes for a FabH homologue, a medium chain fatty acyl-CoA ligase, and a crotonyl-CoA reductase/carboxylase (CCRC), and is predicted to generate the unique 2-alkylmalonyl CoA extender unit for armeniaspirol production [13, 21]. ArmN has also been annotated as an acyl-CoA carboxylase ε-subunit domain, which is known to enhance carboxylase activity and in this biosynthesis, it is proposed to enhance ArmL activity [1, 6]. Consequently, we overexpressed ArmJKLN as a strategy to enhance the 2-alkylmalonyl CoA production (Fig. 1). As a comparison to these earlier two approaches targeting substrate precursors levels, we also examined upregulation of *N*-methyltransferase, ArmO. ArmO is part of the final steps in the biosynthetic pathway [5, 12].

**Fig. 3:**
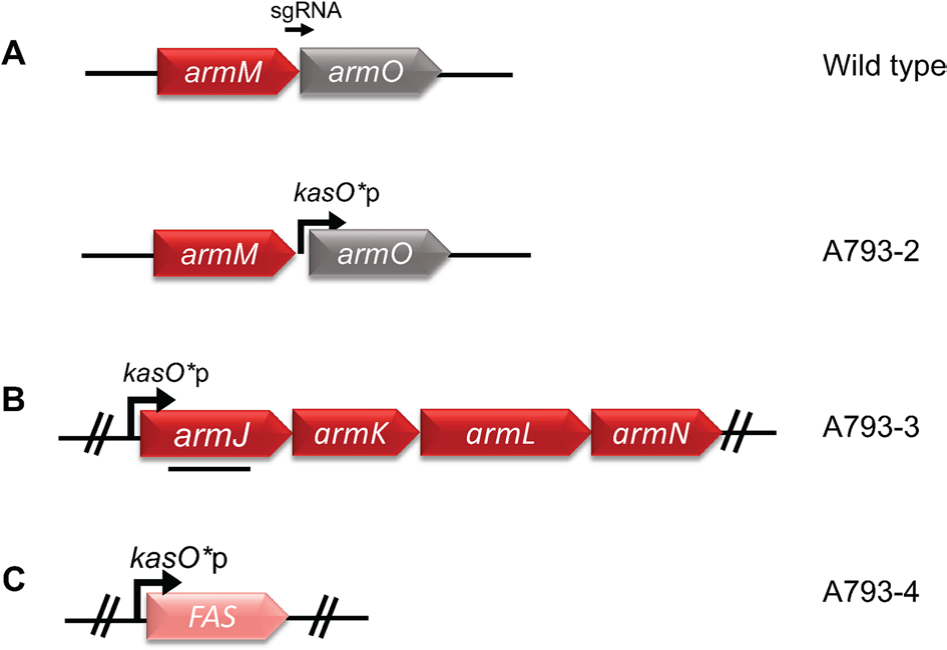
Schematic representations of edits made in this study. These include **A** CRISPR-Cas mediated insertion of a strong constitutive promoter kasO*p in front of *armO* (A793-2) and integrative overexpression cassettes for **B** ArmJKLN (A793-3) and **C** FAS (A793-4)

Due to limitations of strain editing in *Streptomyces* sp. A793, both CRISPR-Cas mediated, and integrative editing techniques were used to realise the above-mentioned engineering strategies (Table 1, Fig. 3, Additional file 1: Fig. S3–6). Mutants were achieved by phiC31 integration of overexpression cassettes for overexpression of ArmJKLN (A793-3) and FAS (A793-4), respectively while CRISPR-Cas mediated insertion of a strong constitutive promoter, kasO*p, in front of the native *armO* was used to upregulate ArmO expression (A793-2).

**Table 1:** Strains used in this study

Strains	Description	Origin
A793 WT	*Streptomyces* sp. A793	[7]
A793-1	Δ*armE*, A793 WT	This work
A793-2	kasO*p-*armO*, A793 WT	This work
A793-3	A793 WT/pSET152-kasO*p_*armJLKN*	This work
A793-4	A793 WT/pSET152-kasO*p-*sco6196*	This work
A793-Δ*nbc20, 21*	Δ*nbc20, 21*, *Streptomyces* sp. A793	[7]
A793-Δ*nbc20, 21–5*	A793-Δ*nbc20, 21*/pSET152-kasO*p-*armJLKN*	This work

### Enhancement in armeniaspirol production

**Fig. 4:**
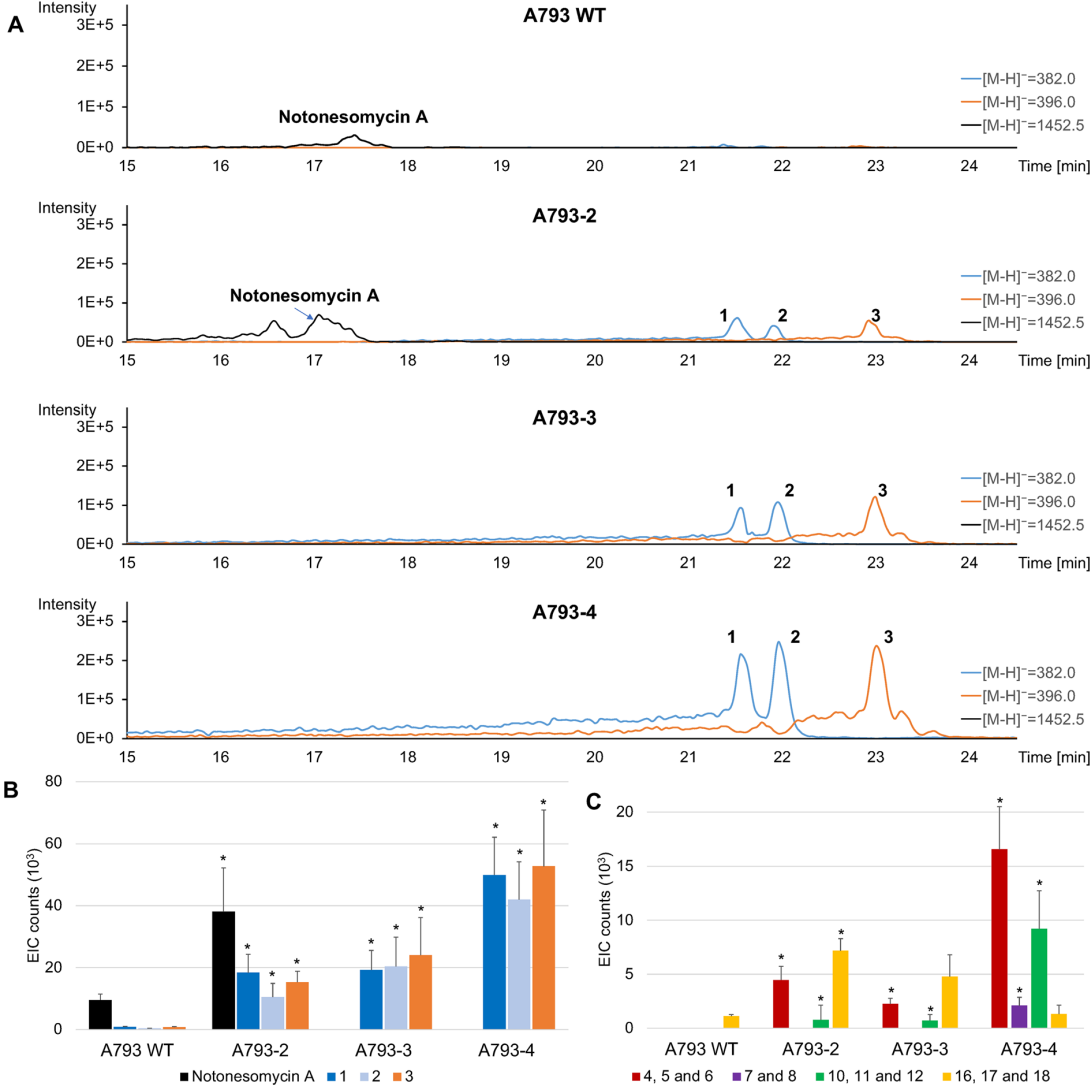
Enhancement of armeniaspirol production. **A** LCMS spectra comparison of notonesomycin A and armeniaspirol production between *Streptomyces* sp. A793 wild type strain (A793 WT) and edited strains A793-2, A793-3 and A793-4. **B** Comparison of the production of armeniaspirols (**1**–**3**) between A793 WT and edited strains A793-2, A793-3 and A793-4. **C** Comparison of the production of armeniaspirols intermediates **4**–**6**, **7**–**8**, **10**–**12** and **16**–**18** between A793 WT and edited strains A793-2, A793-3 and A793-4. **9**, **13**–**15** were not observed in our analyses. Mean values in three independent experiments are presented and error bars refer to standard deviation. Significance of differences to A793 WT was calculated with Student’s *t*-test (* *P*-values < 0.05)

With these three strategies, we observed a significant 12-fold to 97-fold increase in the production of armeniaspirols (**1**–**3**) and in the case of ArmJKLN and FAS overexpressing strains (A793-3 and A793-4, respectively), a suppression in notonesmycin production was also observed (Fig. 4A). FAS overexpressing mutant (A793-4) demonstrated nearly two orders of magnitude increase in armeniaspirol production (97-fold) followed by ArmJKLN (A793-3, 49-fold) and ArmO (A793-2, 12-fold). This suggested that precursors from primary metabolism were indeed limiting armeniaspirol production in this strain under lab conditions (Fig. 4A, B). Not surprisingly, overexpression of FAS, which might be involved in both acyl-CoA and fatty acid biosynthesis pathways, resulted in the highest fold change. Fatty acid products from fatty acid degradation are expected to feed into the extender unit biosynthetic pathway (ArmJKLN) whilst impact on acyl-CoAs is expected to enhance malonyl CoA levels.

Along with the significant increase in armeniaspirols (**1**–**3**) yields, we also observed a corresponding enhancement in intermediates and analogs production in the mutant strains (Fig. 4C). Compared to the WT strain, A793-2 and A793-3 exhibit the presence of previously unobserved mono-chlorinated armeniaspirol analogs (**4**–**6**) and respective precursors **10**–**12**, while **16**–**18** were observed in all three strains. Interestingly, the corresponding tri-chlorinated precursors **13**–**15** or dechlorinated precursors **7**–**9** for armeniaspirols **1**–**3** were not observed in these strains. Based on these observations, we hypothesized that ArmM activity is likely a limiting factor for conversion of **16**–**18** to **10**–**12,** resulting in **16**–**18** accumulation. Moreover, ArmO was known to be specific to doubly chlorinated precursors, **7**–**9** [12]. Consequently, although some analogs **4**–**6** are observed in A793-2 and A792-3, there were small accumulation of the mono-chloro-demethylated armeniaspirols **10**–**12** in A793-2 and A793-3. With overexpression of FAS (A793-4), the ratios of intermediates were altered from earlier observations of A793-2 and A793-3, possibly due to a concurrent increase in reducing equivalents [19]. The increased presence of reducing equivalents may have facilitated ArmM activity and alleviated its bottleneck in converting intermediates **16**–**18** to **10**–**12**. Notwithstanding, the significant increase in flux may have made ArmO the limiting factor leading to appearance of demethylated analogs, **7** and **8**.

## Discussion

Traditional biosynthetic engineering within native strains typically involved significant engineering within the relevant BGCs, assuming that silent or low yielding NPs can be mainly contributed to limited genetic expression towards secondary metabolite production [15, 22]. But in recent years, Wang et al. have shown that balancing this interface of primary and secondary metabolisms is important in ensuring optimal production of secondary metabolites and thus NPs. They also demonstrated that this can be tuned by expression of fatty acyl-CoA synthase (FAS). In this work and prior observations [17], we also observed FAS to be significantly efficient in improving production of silent or low-yielding products. Additionally, similar to observations made previously with notonesomycin production in *Streptomyces* sp. A793 and other prior work [7, 14, 17], we expect that further improvement of yields can be made in regulation of nutrient sources or pleiotropic regulators.

Although the individual engineering strategies had been successful, we were unable to explore them fully due to the genetic inaccessibility of *Streptomyces* sp. A793. However, we did manage to obtain a mutant strain that was integrated with ArmJKLN overexpression cassette in notonesomycin A disrupted strain (A793-Δ*nbc20, 21–5*). This mutant surprisingly gave rise to lower armeniaspirol production (Additional file 1: Figs. S7 and S8). This observation suggested that there are further considerations for metabolic engineering, including the possibility of regulatory effects of notonesomycin or its regulatory elements on armeniaspirols and vice versa [23]. Further in-depth investigation using metabolomics and transcriptomics would be required to elucidate these relationships [24] which might become important factors to consider in heterologous expression of BGCs—local regulatory and feedback regulation.

## Conclusion

To summarize, we demonstrated upregulation of armeniaspirols by up to 97-fold and increased analog diversity through engineering of its native producer. Overexpression of a rare extender unit pathway (ArmJKLN) or even *N*-methyltransferase (ArmO) within the BGC was also sufficient to effect Twelve-fold to Forty nine-fold improvement in armeniaspirol production. However, pleiotropic regulation (via FAS) towards increasing substrate pools of fatty acids and acyl-CoAs led to the most significant fold improvement. Along with upregulation, we also observed a proportional increase in diversity of analogs and intermediates, which will be important in expanding chemical space and optimization of bioactive candidates.

## Materials and methods

### Strains and growth conditions

Strains and plasmids used in this study are listed in Table 1 and Additional file 1: Table S2. Unless otherwise strains are propagated in A793 seed media [4] at 30 °C. Spore preparations and conjugation protocols were similar to those described by [22].

### Construction of editing plasmids

All DNA manipulations were carried out in Escherichia coli OmniMAX™ (Thermo Fisher). The protospacers were initially incorporated into pCRISPRomyces-2 plasmids [18] using BbsI-mediated Golden Gate assembly, followed by the addition of the corresponding homology flanks via Gibson assembly. The methodology for CRISPR plasmid construction was previously detailed [16]. The insertion of kasO*p into CRISPR plasmids were constructed as described in previous study [22]. The integration plasmids were derived by cloning overexpression cassette, under strong constitutive kasO*p promoter, into pSET152 (Additional file 1: Table S2).

### Genome editing

DNA methylation-proficient WM6026 *E.coli* strains were used to perform conjugation experiments with R2 agar without sucrose. CRISPR-Cas mediated editing protocols were similar as described before [22]. For integration with pSET152 derived plasmids, apramycin selection was used to select for mutants. PCR was performed using genomic DNA extracted from exconjugants to screen for edited strains and positive samples were sent for Sanger sequencing.

### Cultivation conditions

Seed culture was grown in seed media (0.4% glucose, 0.4% yeast extract, 1% malt extract, 0.2% CaCO_3_, pH 7.0) incubated for 3 days at 28 °C, 200 rpm. 5% seed culture was used to inoculate shake flasks fermentation. Strains were fermented in ISP2 (0.4% glucose, 0.4% yeast extract, 1% malt extract, pH 7.0) for 9 days at 28 °C, 200 rpm.

### Sample preparation and LCMS analysis

The supernatant was lyophilized, extracted with methanol (1.6 mL), disrupted by sonication and centrifuged to remove the cell debris. The methanol supernatant (40 µL) was analysed by HPLC–MS (Agilent, single quadrupole G6120B and QTOF 6545B) equipped with Acquity Premier UPLC BEH C18 column (50 × 2.1 mm, 1.7 μm, 130 Å). HPLC parameters were as follows: solvent A, 0.1% formic acid in water; solvent B, 0.1% formic acid in acetonitrile; gradient at a constant flow rate of 0.6 mL/min, 5 to 95% B in 37 min, 95% B for 3 min, 95 to 5% B in 1 min, 5% B for 4 min. Identity of armeniaspirols (**1**–**3**) was determined by comparison with authentic samples (Additional file 1: Fig. S9). HRMS was also utilized for identification of intermediates and compounds (Additional file 1: Figs. S10–17). The estimated yield (based on MS peak area of authentic sample) for Armeniaspirol A, B and C is 2.7 mg/L, 1.9 mg/L and 2.3 mg/L respectively in A793-4.

## Supplementary Information

**Additional file 1: Figure S1.** Comparison of the production armeniaspirols (**1**-**3**) between A793 WT and notonesomycin BGC disrupted strain (A793-∆*nbc20, 21*).** Figure S2.** *Streptomyces* sp. A793 with *armE* deletion. **Figure S3.** Schematic of deletion of *nbc20, 21* for disruption of notonesomycin production. **Figure S4**. *Streptomyces* sp. A793 with kasO*p insertion before *armO*. **Figure S5**. PCR verification of integrated kasO*p-*armJKLN* cassette plasmid in *Streptomyces* sp. A793 genome. **Figure S6. **PCR verification of integrated kasO*p-*sco6196* cassette plasmid in *Streptomyces* sp. A793 genome. **Figure S7**. LCMS analysis of A793 WT, A793-Δ*nbc20, 21 *and A793-Δ*nbc20, 21*-5. **Figure S8. **PCR verification of integrated kasO*p-*armJKLN *cassette plasmid in *Streptomyces* sp. A793*,* ∆*nbc20, 21* genome. **Figure S9.** LCMS analysis of authentic samples of **1**, **2 **and **3**. **Figure S10.** HPLC-MS/MS fragmentation analysis of **1**-**6**. **Figure S11.** HPLC-MS/MS fragmentation analysis of **7**-**12**. **Figure S12.** HPLC-MS/MS fragmentation analysis of **16**-**18**. **Figure S13.** The comparisons of observed isotope pattern and calculated isotope pattern of Notonesomycin A, **1**, **2** and **3**. **Figure S14.** The comparisons of observed isotope pattern and calculated isotope pattern of **4**, **5** and **6**. **Figure S15.** The comparisons of observed isotope pattern and calculated isotope pattern of **7** and **8**. **Figure S16.** The comparisons of observed isotope pattern and calculated isotope pattern of **10**, **11** and **12**. **Figure S17.** The comparisons of observed isotope pattern and calculated isotope pattern of **16**, **17** and **18**. **Table S1. **Armeniaspirol biosynthetic gene cluster in *Streptomyces* sp. A793 **Table S2. **List of plasmids used in this study.

## Data Availability

The data supporting the conclusions of this article are included within the article and its supplementary data.

## Abbreviations

NP: Natural product
BGC: Biosynthetic gene cluster
FAS: Fatty acyl-CoA synthase
CCRC: Crotonyl-CoA reductase/carboxylase
CRISPR: Clustered regularly interspaced short palindromic repeats
*E.coli*: *Escherichia coli*
HRMS: High-resolution mass spectrometry
