# Supplementary material for: Enhancing armeniaspirols production through multi-level engineering of a native Streptomyces producer
Source: Microb Cell Fact. 2023 Apr 28;22:84. doi: 10.1186/s12934-023-02092-4 (PMC10142417; doi:10.1186/s12934-023-02092-4)
Supplement: Supplementary file 1 — Additional file 1: Figure S1. Comparison of the production armeniaspirols (1-3) between A793 WT and notonesomycin BGC disrupted strain (A793-∆nbc20, 21). Figure S2. Streptomyces sp. A793 with armE deletion. Figure S3. Schematic of deletion of nbc20, 21 for disruption of notonesomycin production. Figure S4. Streptomyces sp. A793 with kasO*p insertion before armO. Figure S5. PCR verification of integrated kasO*p-armJKLN cassette plasmid in Streptomyces sp. A793 genome. Figure S6. PCR verification of integrated kasO*p-sco6196 cassette plasmid in Streptomyces sp. A793 genome. Figure S7. LCMS analysis of A793 WT, A793-Δnbc20, 21 and A793-Δnbc20, 21-5. Figure S8. PCR verification of integrated kasO*p-armJKLN cassette plasmid in Streptomyces sp. A793, ∆nbc20, 21 genome. Figure S9. LCMS analysis of authentic samples of 1, 2 and 3. Figure S10. HPLC-MS/MS fragmentation analysis of 1-6. Figure S11. HPLC-MS/MS fragmentation analysis of 7-12. Figure S12. HPLC-MS/MS fragmentation analysis of 16-18. Figure S13. The comparisons of observed isotope pattern and calculated isotope pattern of Notonesomycin A, 1, 2 and 3. Figure S14. The comparisons of observed isotope pattern and calculated isotope pattern of 4, 5 and 6. Figure S15. The comparisons of observed isotope pattern and calculated isotope pattern of 7 and 8. Figure S16. The comparisons of observed isotope pattern and calculated isotope pattern of 10, 11 and 12. Figure S17. The comparisons of observed isotope pattern and calculated isotope pattern of 16, 17 and 18. Table S1. Armeniaspirol biosynthetic gene cluster in Streptomyces sp. A793 Table S2. List of plasmids used in this study. [file 12934_2023_2092_MOESM1_ESM.pdf]

Enhancing armeniaspirols production through multi-level engineering of a native *Streptomyces* producer

Elena Heng,[a*] Yi Wee Lim,[b*] Chung Yan Leong,[c] Veronica W. P. Ng,[c], Siew Bee Ng,[c] Yee Hwee Lim,[b#] Fong Tian Wong[a,b#]

* These authors contributed equally.

Affiliations:

[a] Molecular Engineering Laboratory, Institute of Molecular and Cell Biology (IMCB), Agency for Science, Technology and Research (A*STAR), 61 Biopolis Drive, #07-06, Proteos, Singapore 138673, Singapore

[b] Chemical Biotechnology and Biocatalysis, Institute of Sustainability for Chemicals, Energy and Environment (ISCE^2), Agency for Science, Technology and Research (A*STAR), 8 Biomedical Grove, Neuros, #07-01, Singapore 138665, Singapore.

[c] Singapore Institute of Food and Biotechnology Innovation (SIFBI), Agency for Science, Technology and Research (A*STAR), 31 Biopolis Way, Level 2, Nanos, Singapore 138669, Singapore

[#]Corresponding authors:

Y.H.L.: lim_yee_hwee@isce2.a-star.edu.sg

F.T.W.: wongft@imcb.a-star.edu.sg

Supplementary information

Figure S1. Comparison of the production armeniaspirols (**1-3)** between A793 WT and notonesomycin BGC disrupted strain (A793-Δ*nbc20, 21*).
Figure S2. *Streptomyces* sp. A793 with *armE* deletion
Figure S3. Schematic of deletion of *nbc20, 21* for disruption of notonesomycin production
Figure S4. *Streptomyces* sp. A793 with *kasO**p insertion before *armO*
Figure S5. PCR verification of integrated *kasO**p-*armJKLN* cassette plasmid in *Streptomyces* sp. A793 genome
Figure S6. PCR verification of integrated *kasO**p-*sco6196* cassette plasmid in *Streptomyces* sp. A793 genome
Figure S7. LCMS analysis of A793 WT, A793-Δ*nbc20, 21* and A793-Δ*nbc20, 21*-5.
Figure S8. PCR verification of integrated *kasO**p-*armJKLN* cassette plasmid in *Streptomyces* sp. A793*, Δnbc20, 21* genome
Figure S9. LCMS analysis of authentic samples of **1**, **2** and **3**.
Figure S10. HPLC-MS/MS fragmentation analysis of **1**-**6**.
Figure S11. HPLC-MS/MS fragmentation analysis of **7**-**12**.
Figure S12. HPLC-MS/MS fragmentation analysis of **16**-**18**.
Figure S13. The comparisons of observed isotope pattern and calculated isotope pattern of Notonesomycin A, **1**, **2** and **3**.
Figure S14. The comparisons of observed isotope pattern and calculated isotope pattern of **4**, **5** and **6**.
Figure S15. The comparisons of observed isotope pattern and calculated isotope pattern of **7** and **8**.
Figure S16. The comparisons of observed isotope pattern and calculated isotope pattern of **10**, **11** and **12**.
Figure S17. The comparisons of observed isotope pattern and calculated isotope pattern of **16**, **17** and **18**.
Table S1. Armeniaspirol biosynthetic gene cluster in *Streptomyces* sp. A793
Table S2. List of plasmids used in this study

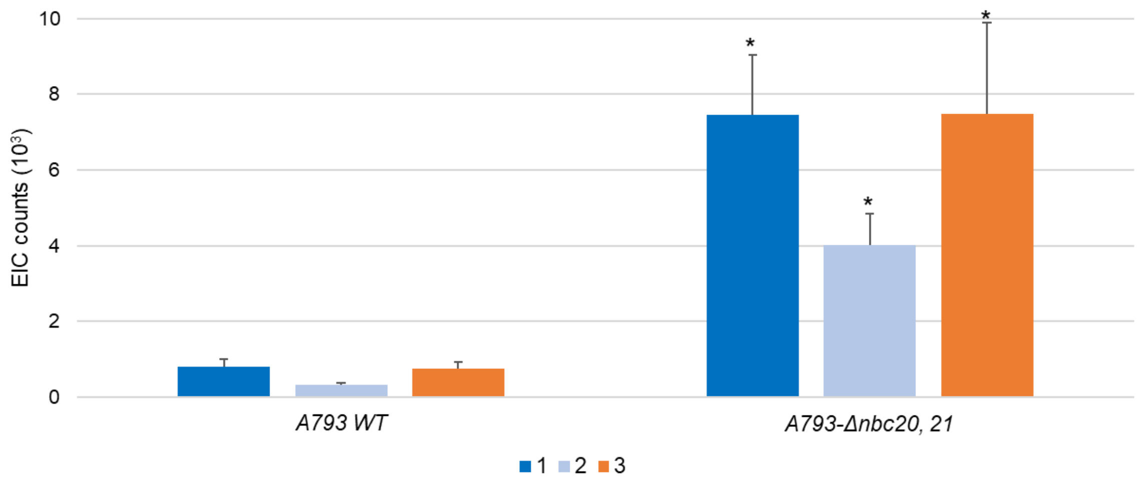

Figure S1. Comparison of the production armeniaspirols (**1**-**3)** between A793 WT and notonesomycin BGC disrupted strain (A793-Δ*nbc20, 21*). Mean values in three independent experiments are presented and error bars refer to standard deviation. Significance of differences to A793 WT was calculated with Student's *t*-test (* *P*-values <0.05).

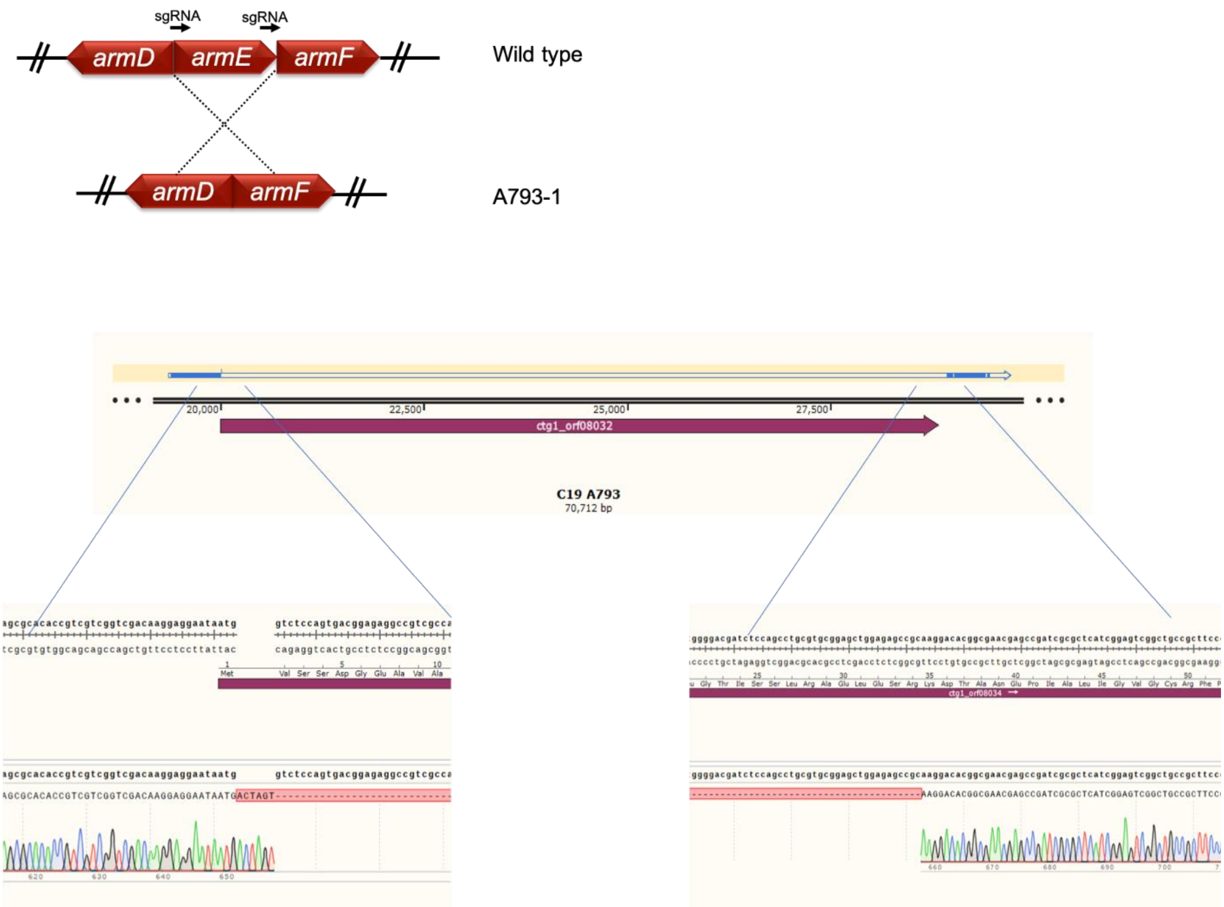

Figure S2. *Streptomyces* sp. A793 with *armE* deletion. Sequence alignment of edited genome against wild type is shown here.

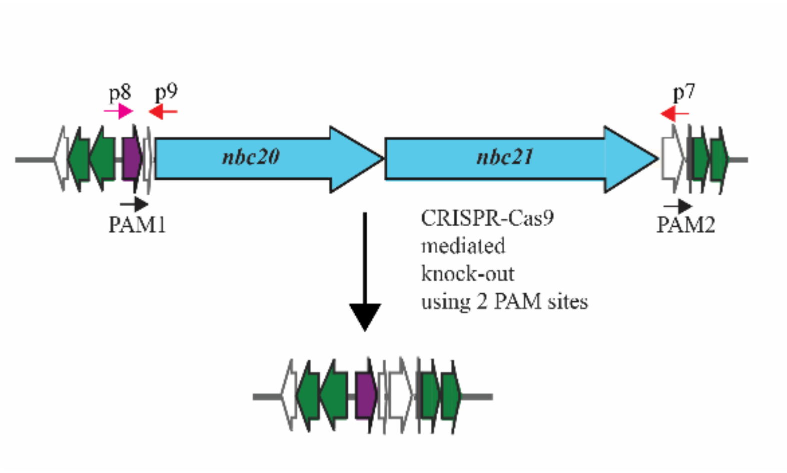

Figure S3. Schematic of deletion of *nbc20, 21* for disruption of notonesomycin production (Figure has been adapted from Goh et al., 2019)

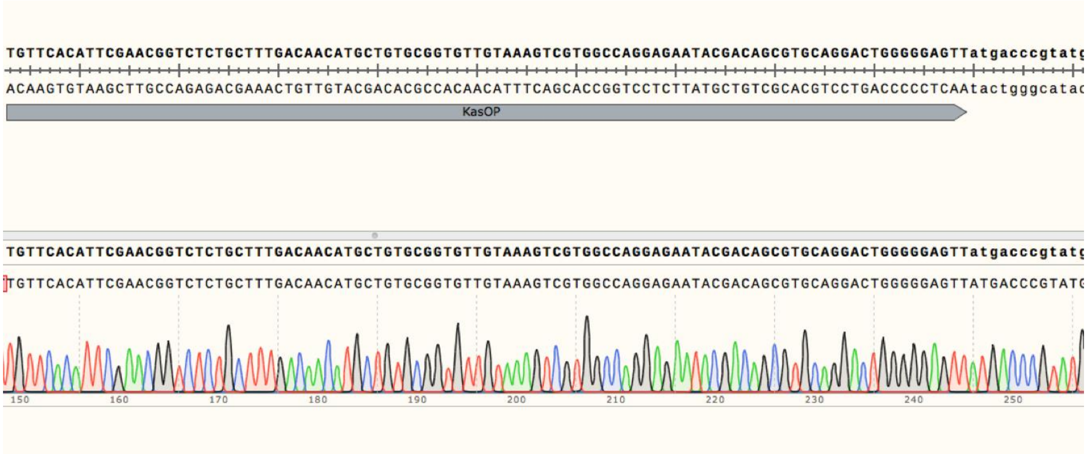

Figure S4. *Streptomyces* sp. A793 with *kasO**p insertion before *armO.* Sequence alignment against *kasO**p is shown.

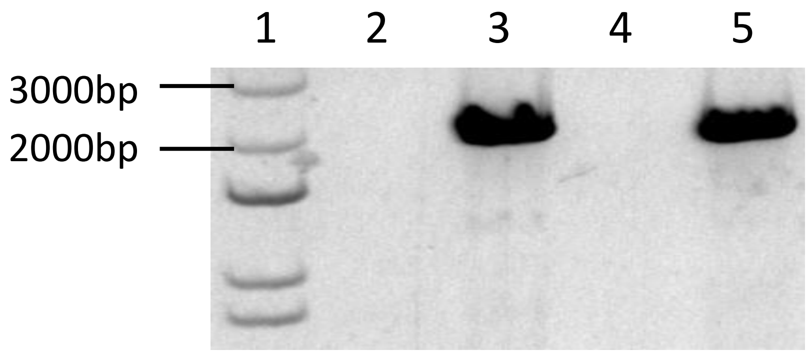

Figure S5. PCR verification of integrated *kasO**p-*armJKLN* cassette plasmid in *Streptomyces* sp. A793 genome using armL flanking primers. 1) NEB Quick-Load 1 kb Extend DNA Ladder, 2) negative control, 3) *kasO**p-*armJKLN* cassette plasmid, 4) Wild-type *Streptomyces* sp. A793 genome, 5) A793-3. Sanger sequencing was also performed to verify the insertion.

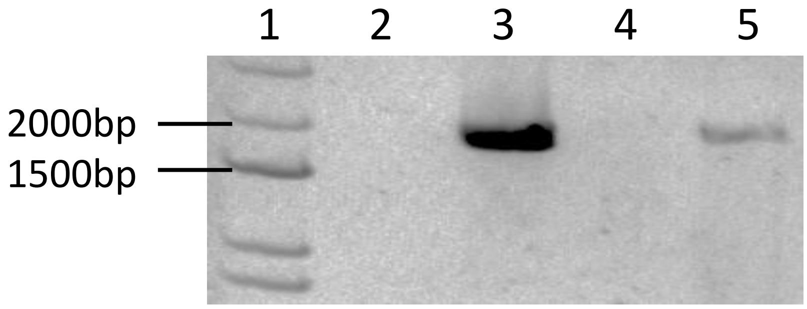

Figure S6. PCR verification of integrated *kasO**p-*sco6196* cassette plasmid in *Streptomyces* sp. A793 genome using *SCO6196* flanking primers. 1) NEB Quick-Load 1 kb Extend DNA Ladder, 2) negative control, 3) *kasO**p-*sco6196* cassette plasmid, 4) Wild-type *Streptomyces* sp. A793 genome, 5) A793-4. Sanger sequencing was also performed to verify the insertion.

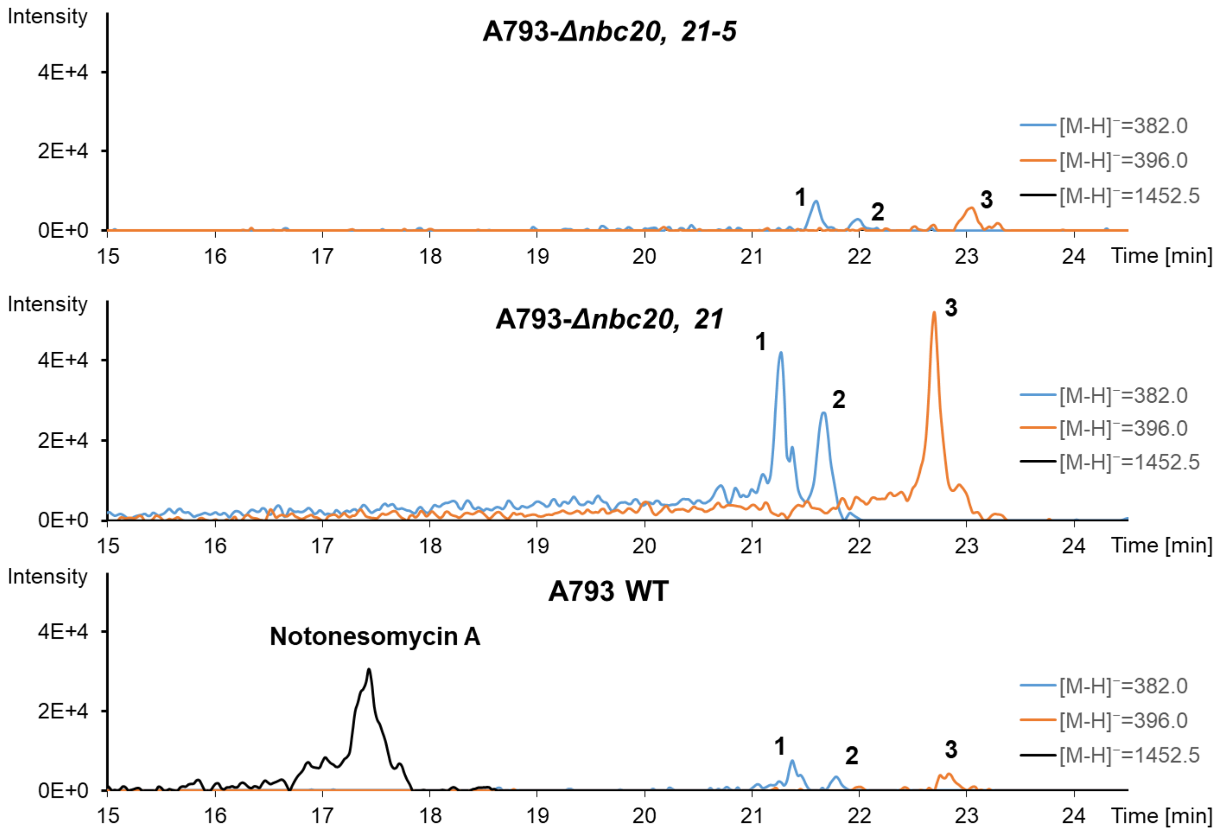

Figure S7. LCMS analysis of A793 WT, A793-Δ*nbc20, 21* and A793-Δ*nbc20, 21*-5. Extracted ion chromatograms (m/z 382, 396, 1452.5) are shown. Notonesomycin A and compounds **1**-**3** are annotated on the spectra.

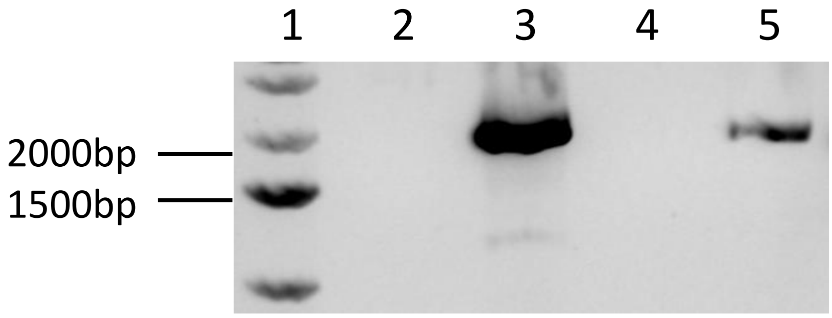

Figure S8. PCR verification of integrated *kasO**p-*armJKLN* cassette plasmid in *Streptomyces* sp. A793, Δ*nbc20, 21* genome using armL flanking primers. 1) NEB Quick-Load 1 kb Extend DNA Ladder, 2) negative control, 3) *kasO**p-*armJKLN* cassette plasmid, 4) *Streptomyces* sp. A793, Δ*nbc20, 21* genome, 5) A793-5. Sanger sequencing was also performed to verify the insertion.

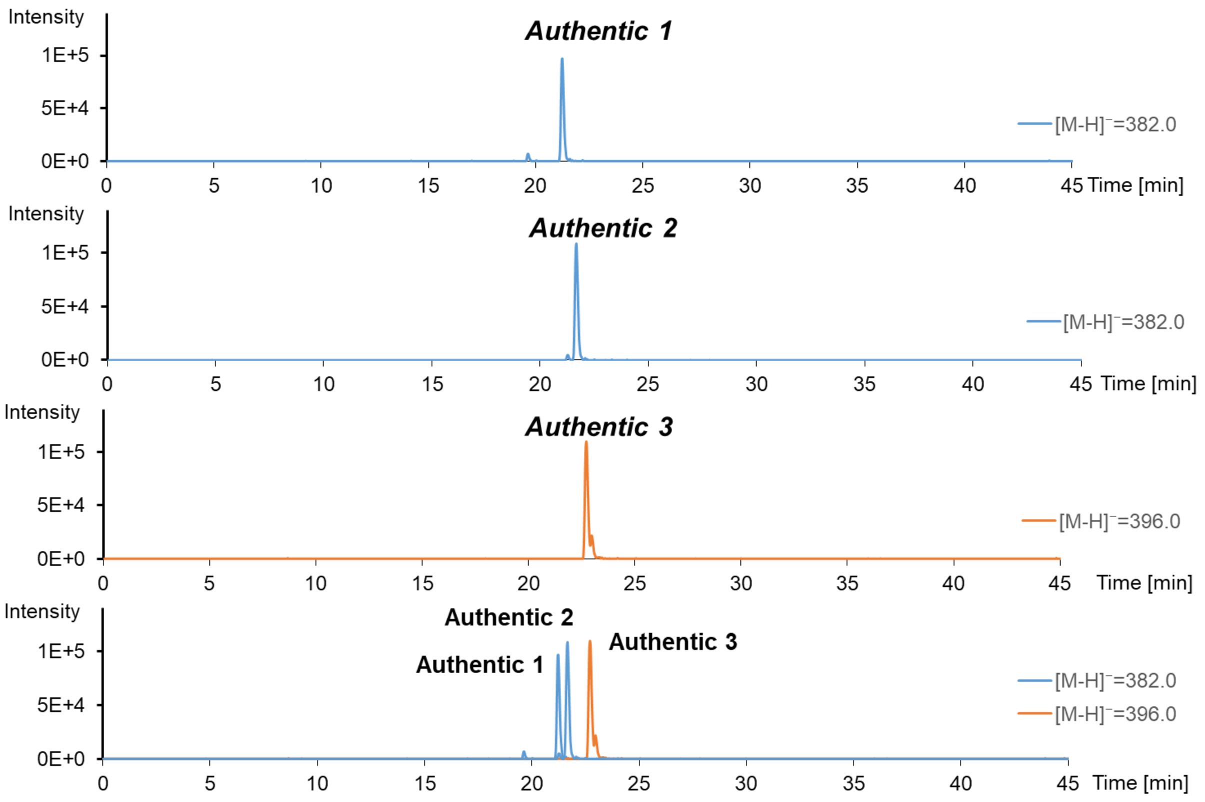

.

Figure S9. LCMS analysis of authentic samples of **1**, **2** and **3** and overlay of the 3 authentic compounds. Extracted ion chromatograms (m/z 382, 396) are shown. Authentic samples were obtained from the Natural Organism Library (Ng et al., 2018), A*STAR, Singapore.

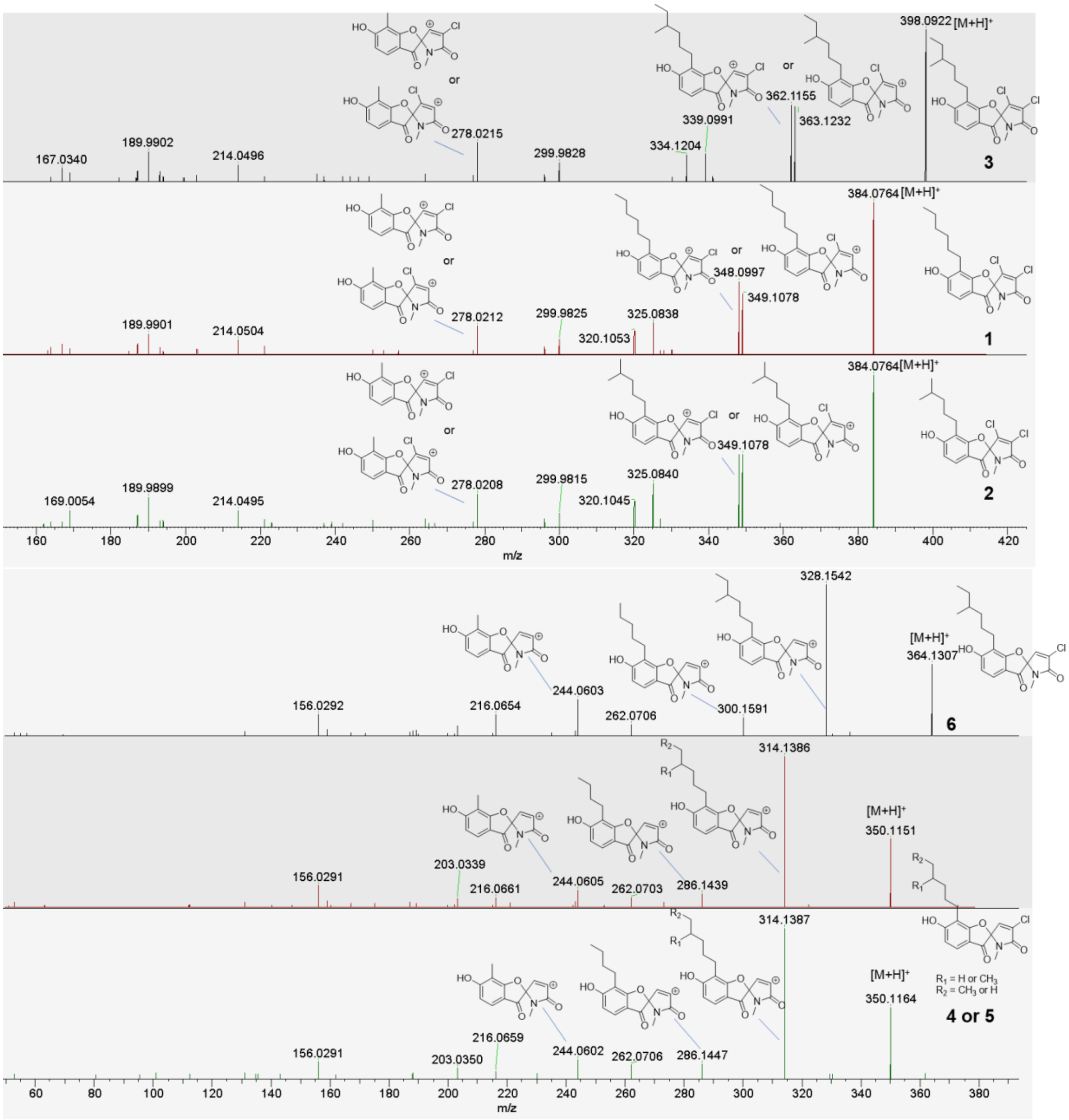

Figure S10. HPLC-MS/MS fragmentation analysis of compounds **1-6**. This is in agreement with the results reported in Fu et al. 2019.

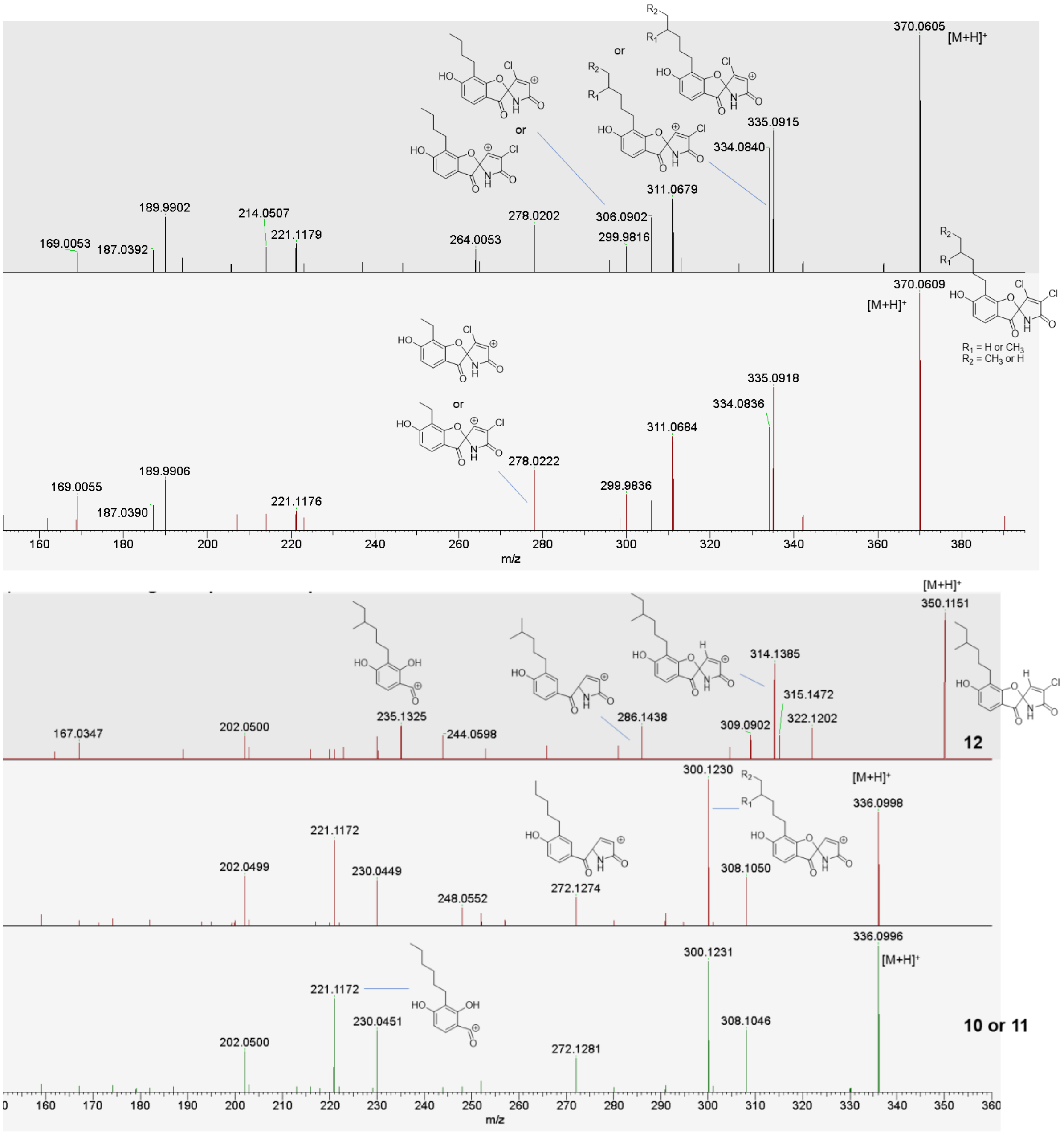

Figure S11. HPLC-MS/MS fragmentation analysis of compounds **7**-**12**. This is in agreement with the results reported in Fu et al. 2019.

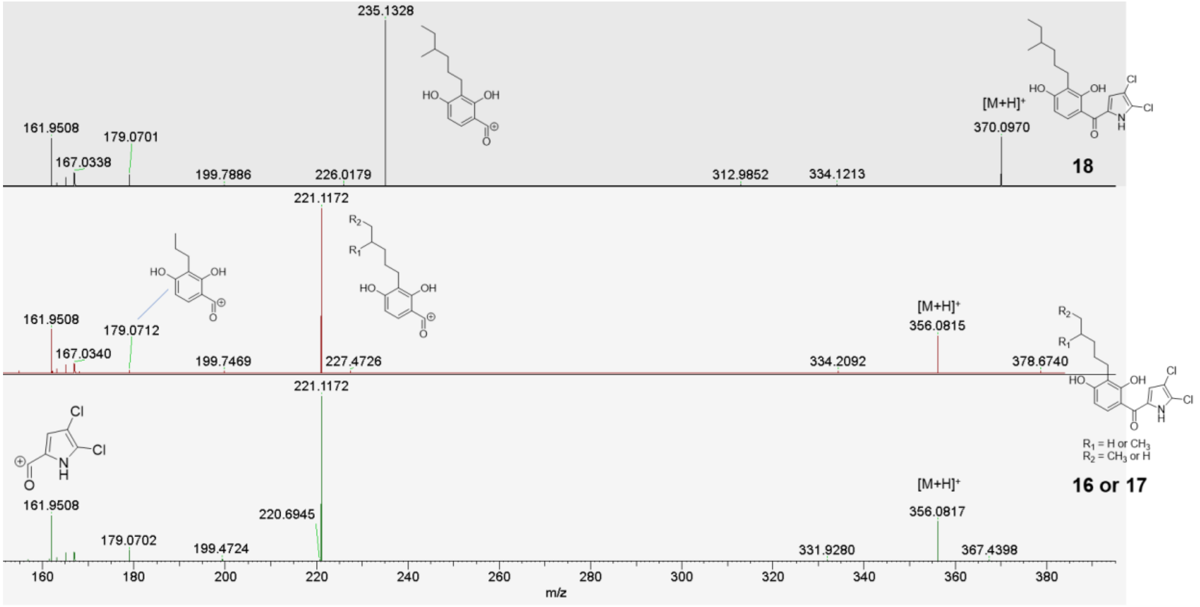

Figure S12. HPLC-MS/MS fragmentation analysis of compounds **16-18**. This is in agreement with the results reported in Fu et al. 2019 paper.

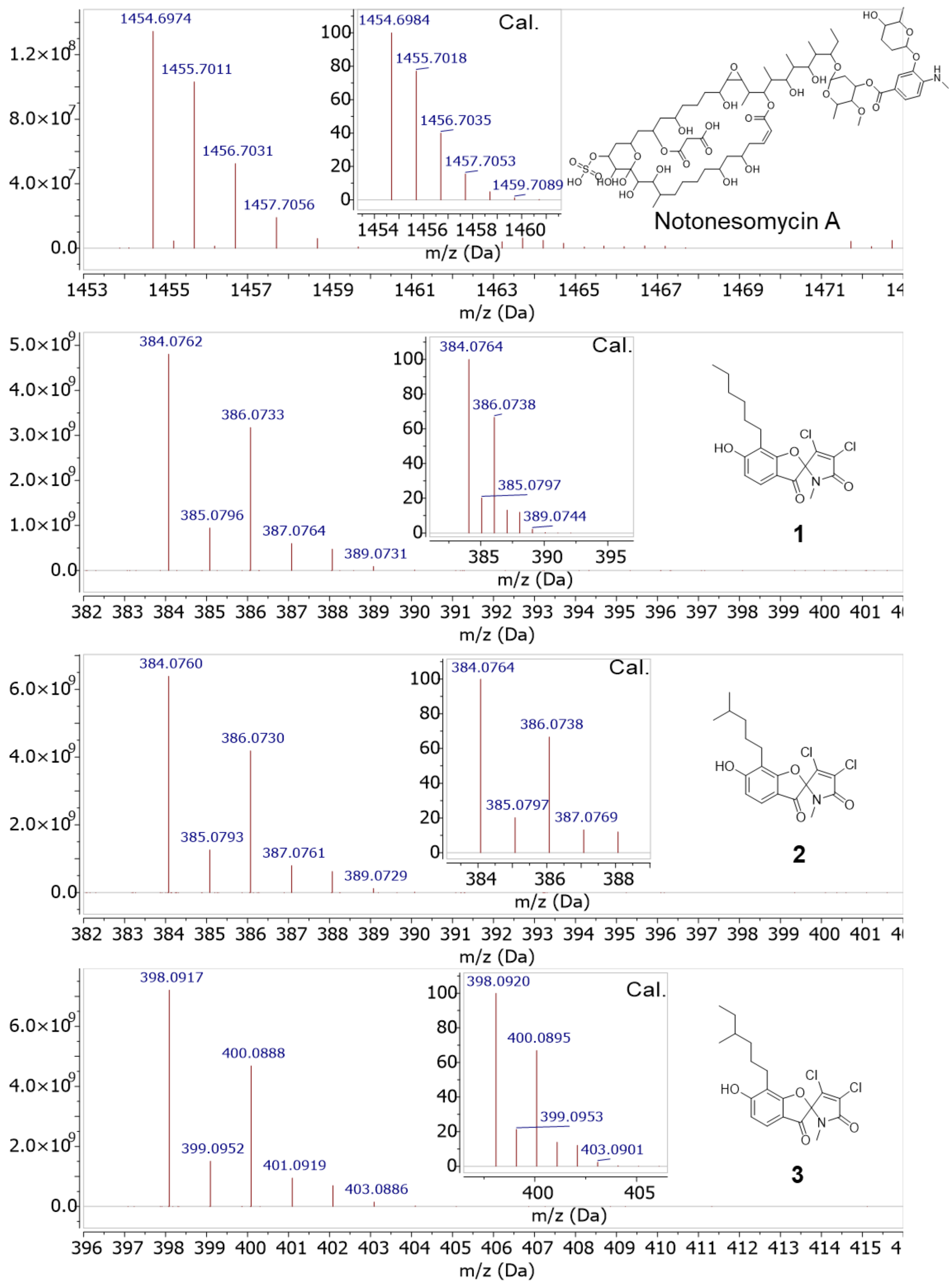

Figure S13. Comparisons of HRMS observed isotope pattern and calculated isotope pattern of Notonesomycin A, **1**, **2** and **3**.

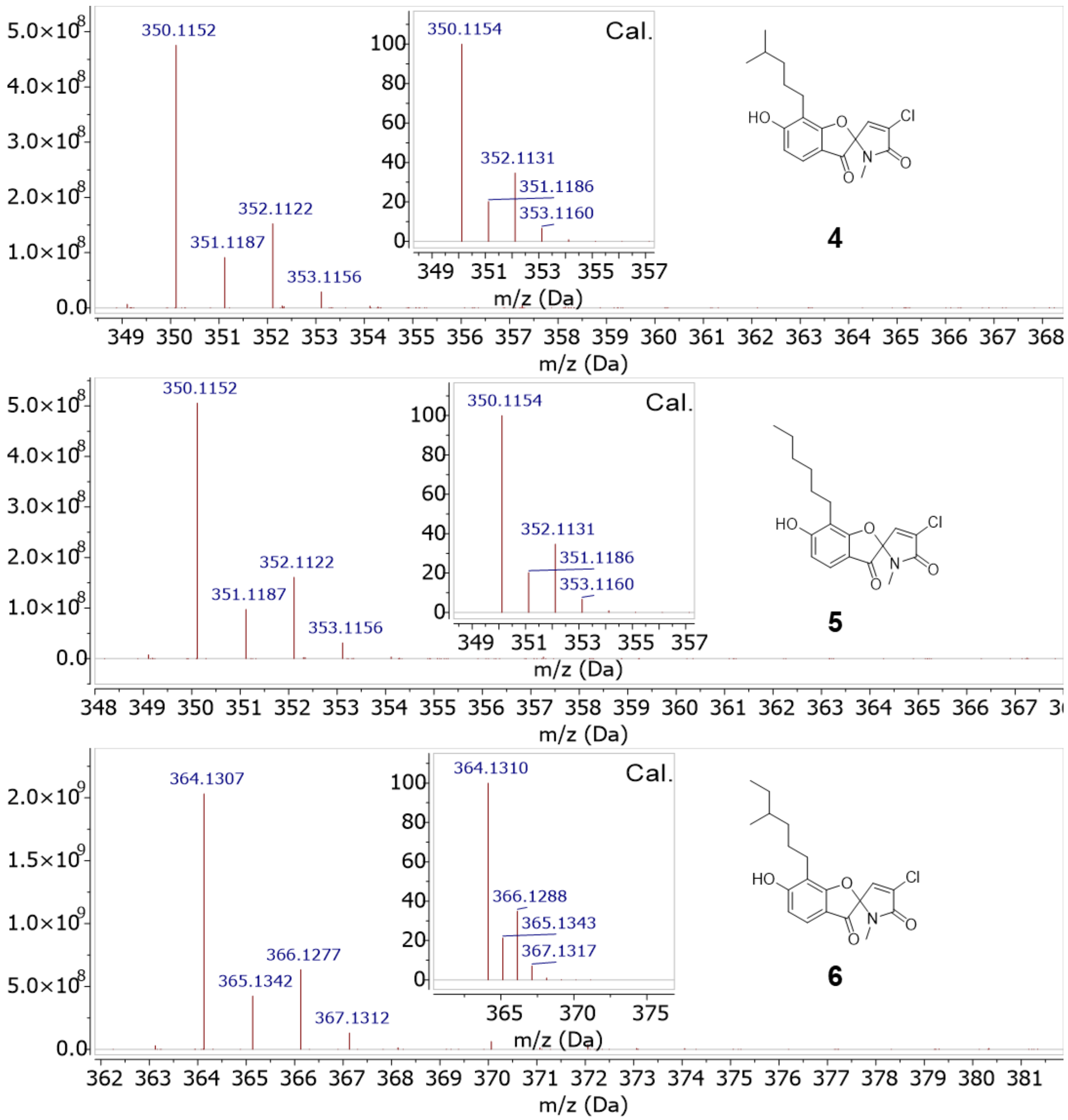

Figure S14. Comparisons of HRMS observed isotope pattern and calculated isotope pattern of **4**, **5** and **6**.

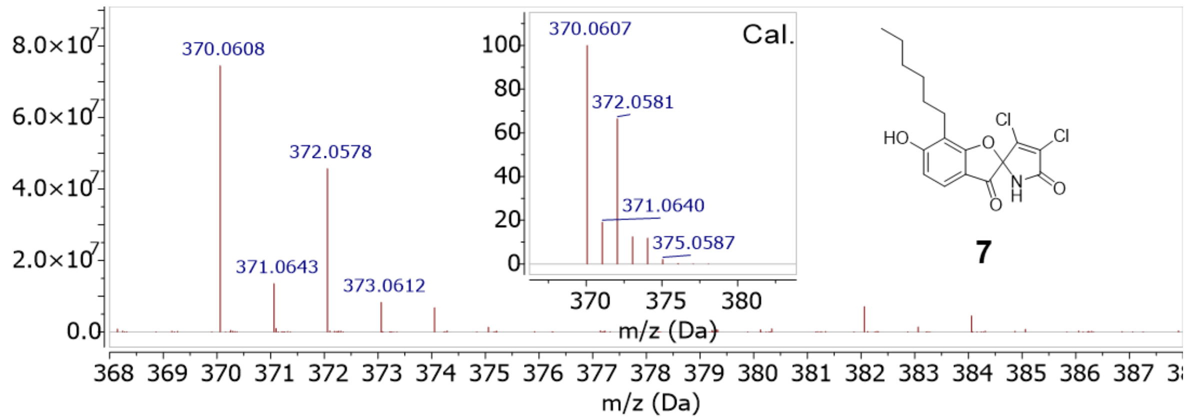

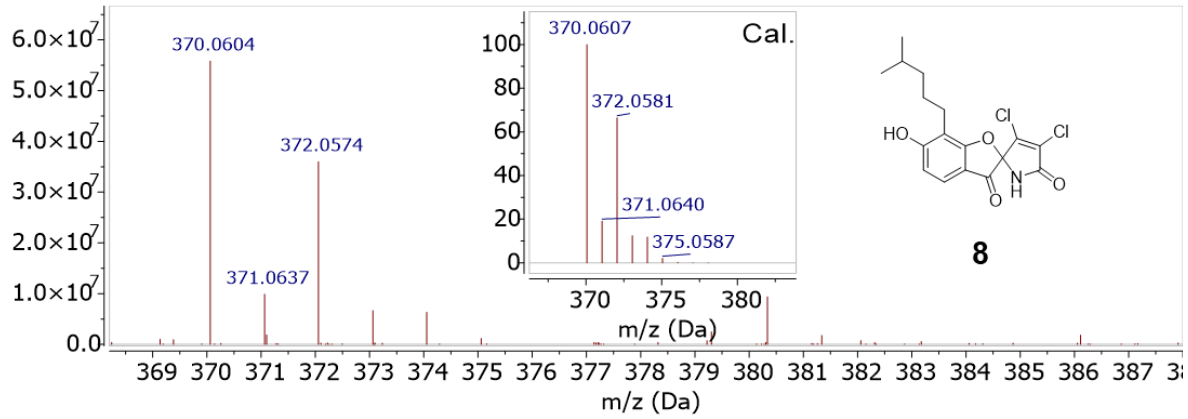

Figure S15. Comparisons of HRMS observed isotope pattern and calculated isotope pattern of **7** and **8**.

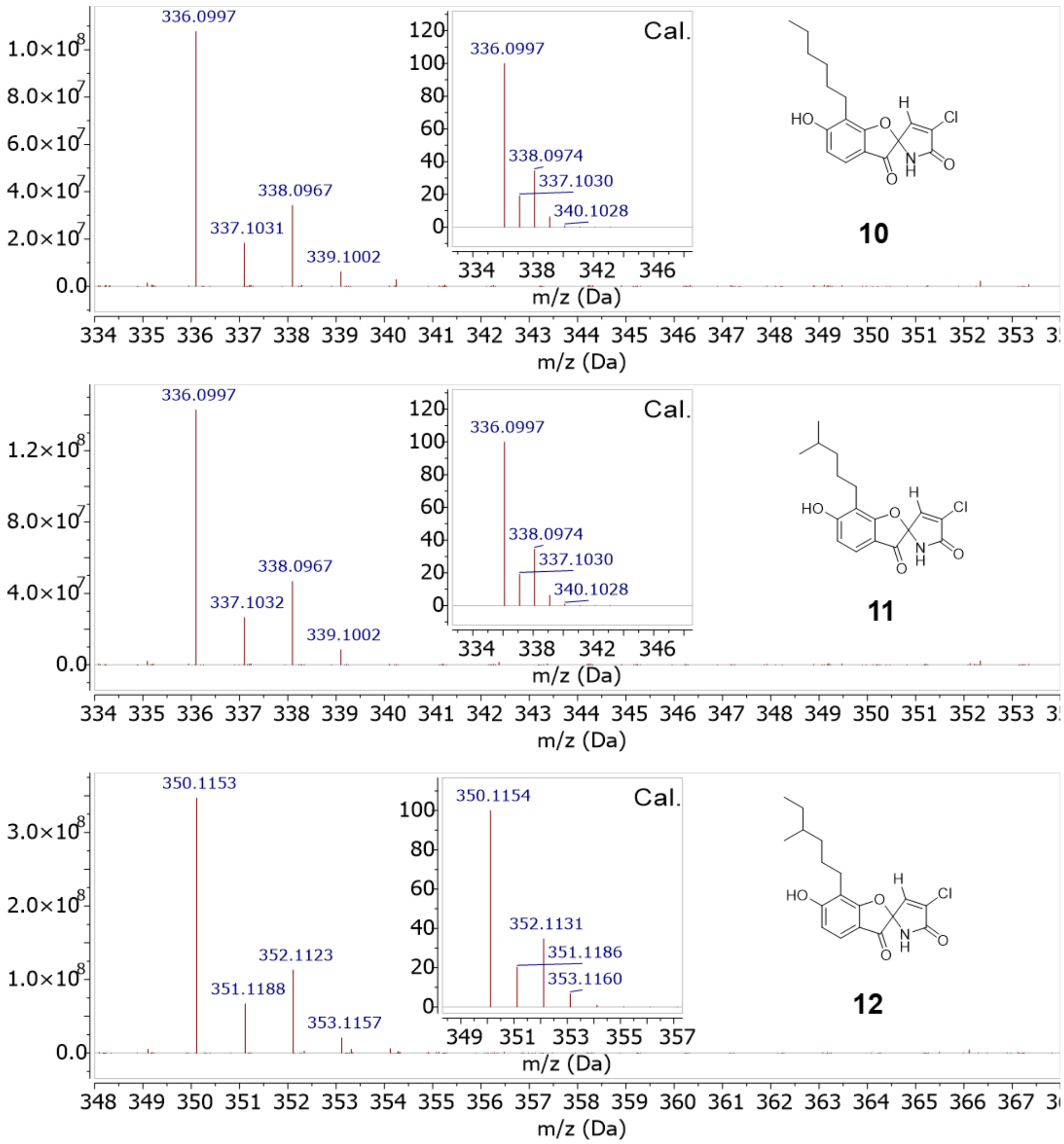

Figure S16. Comparisons of HRMS observed isotope pattern and calculated isotope pattern of **10**, **11** and **12**.

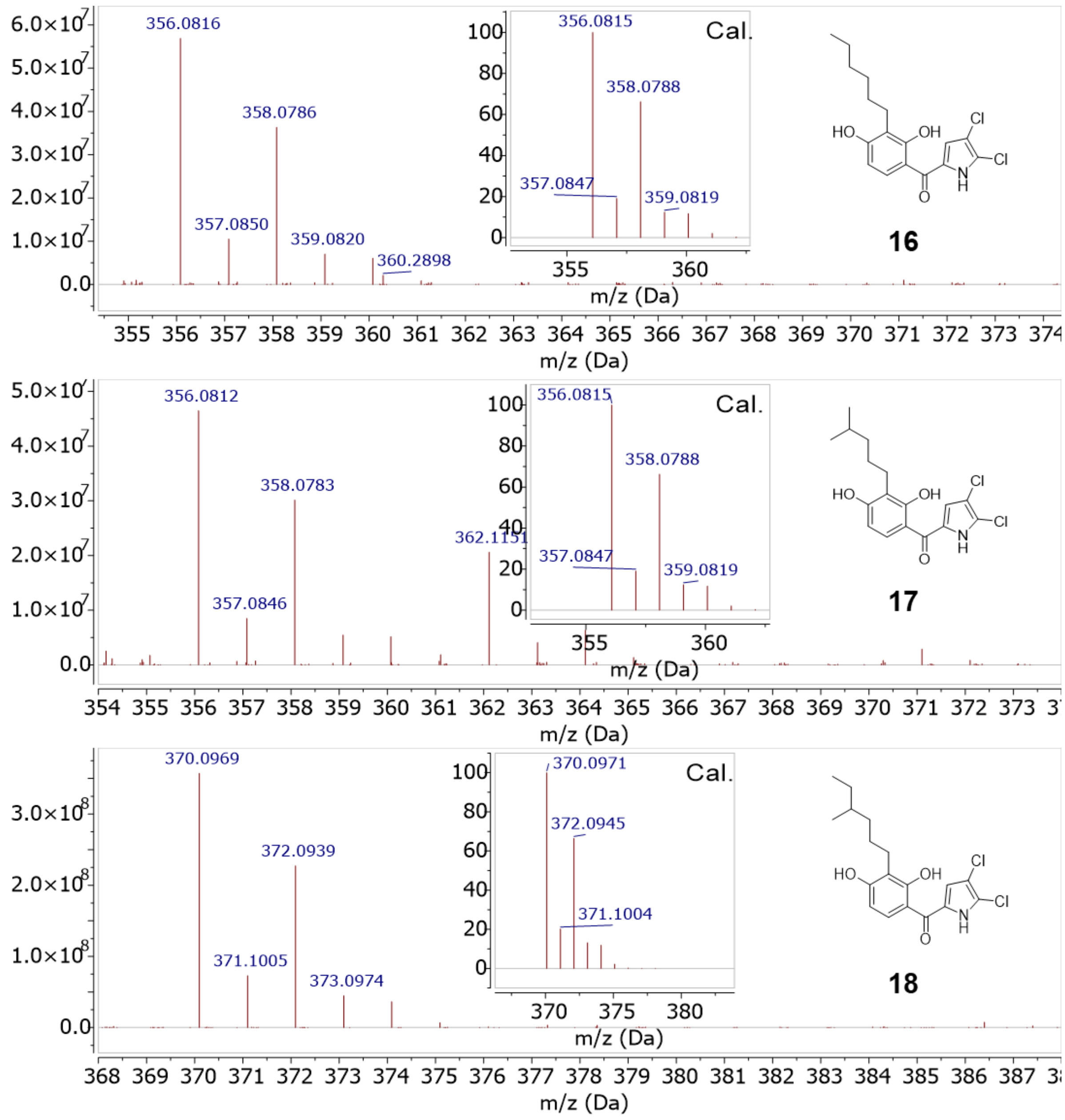

Figure S17. Comparisons of HRMS observed isotope pattern and calculated isotope pattern of **16**, **17** and **18**.

Table S1. Armeniaspirol gene cluster from A793, compared to miBIG BGC0002022: armeniaspirol A biosynthetic gene cluster from *Streptomyces armeniacus*. Gene arrangement for armeniaspirol; blue represents transporter-related genes, red represents core biosynthetic genes, green represents regulatory genes and grey represents unknown genes.

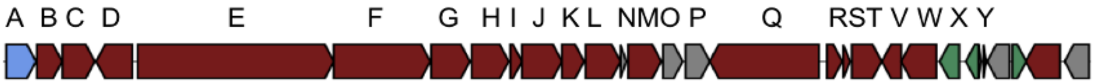

Label	Location	Homolog	Coverage, similarity (%)	Coresponding gene in BGC	Percent identity (%)
armA	ctg1_orf08023	membrane antiporter [Streptomyces sp. UC 11065]	92, 59	*ams2*	100
armB	ctg1_orf08026	acyl-CoA carboxylase subunit beta [Streptomyces sp. NBRC 109706]	99, 79	*ams3*	99.74
armC	ctg1_orf08027	amino acid adenylation domain-containing protein [Streptomyces sp. MP131-18]	100, 72	*ams4*	100
armD	ctg1_orf08029	PrlM [Nonomuraea spiralis]	98, 50	*ams5*	100
armE	ctg1_orf08032	type I polyketide synthase [Streptomyces sp. MP131-18]	98, 62	*ams6*	100
armF	ctg1_orf08034	type I polyketide synthase [Streptomyces sp. MP131-18]	99, 68	*ams7*	100
armG	ctg1_orf08037	NAD(P)/FAD-dependent oxidoreductase [Streptomyces davaonensis]	98, 56	*ams8*	100
armH	ctg1_orf08038	halogenase B [Streptomyces davaonensis]	97, 75	*ams9*	99.82
armI	ctg1_orf08040	flavin reductase [Streptomyces alni]	99, 59	*ams10*	100
armJ	ctg1_orf08041	fatty acyl-AMP ligase [Streptomyces aidingensis]	97, 66	*ams11*	100
armK	ctg1_orf08042	beta-ketoacyl synthase III [Streptomyces griseoruber]	96, 66	*ams12*	99.7
armL	ctg1_orf08043	acyl-CoA carboxylase subunit beta [Streptomyces sp. NBRC 109706]	97, 81	*ams13*	99.81
armN	ctg1_orf08045	acyl-CoA carboxylase subunit epsilon [Arthrobacter psychrochitiniphilus]	75,42	*ams14*	98.82
armM	ctg1_orf08047	monooxygenase, FAD-binding [Actinomadura parvosata subsp. kistnae]	99, 59	*ams15*	99.8
armO	ctg1_orf08050	SAM-dependent methyltransferase [Streptosporangium roseum]	97, 53	*ams16*	100
armP	ctg1_orf08051	IS630 family transposase [Streptomyces sp. BSE7F]	99, 93	*ams17*	99.72
armQ	ctg1_orf08053	type I polyketide synthase [Streptomyces vitaminophilus]	99, 52	*ams19*	99.94
armR	ctg1_orf08054	S-acyl fatty acid synthase thioesterase [Streptomyces vitaminophilus]	87, 64	*ams20*	100
armS	ctg1_orf08056	proline carrier protein [Streptomyces vitaminophilus]	80, 68	*ams21*	98.88

armT	ctg1_orf08057	NAD(P)/FAD-dependent oxidoreductase [Streptomyces sp. MP131-18]	99, 87	*ams22*	99.78
armU	ctg1_orf08058	4'-phosphopantetheinyl transferase superfamily protein [Streptomyces sp. CB02959]	92, 47	*ams23*	99.66
armV	ctg1_orf08060	acyl-CoA dehydrogenase [Streptomyces sp. IMTB 2501]	92, 39	*ams24*	100
armW	ctg1_orf08061	SARP family transcriptional regulator [Streptomyces laurentii]	85, 65	*ams25*	99.64
armX	ctg1_orf08064	DNA-binding response regulator [Streptomyces angustmyceticus]	98, 68	*ams26*	100
armY	ctg1_orf08068	TetR/AcrR family transcriptional regulator [Streptomyces sp. TAA486]	95, 75	*ams28*	100
orf1	ctg1_orf08069	acyl--CoA ligase [Streptomyces hygroscopicus]	98, 56	AZY91996.1	100
orf2	ctg1_orf08070	winged helix DNA-binding domain-containing protein [Streptomyces sp. TAA486]	98, 62	AZY92006.1	100

Table S2. List of plasmids used in this study

Plasmids	Description	Origin
pCRP4	pCRISPRomyces-*armE* PS, *armE* homology template for deletion	This work
pCRP14	pCRISPRomyces-*kasO**p-*armO* PS, *armO* homology template for insertion	This work
pCRP178	pSET152- *kasO**p-*sco6196*	This work
pCRP186	pSET152- *kasO**p-*armJKLN* cassette	This work

References:

Goh F, Zhang MM, Lim TR, et al (2020) Identification and engineering of 32 membered antifungal macrolactone notonesomycins. Microb Cell Fact 19:71. https://doi.org/10.1186/s12934-020-01328-x

Qiao Y, Yan J, Jia J, et al (2019) Characterization of the Biosynthetic Gene Cluster for the Antibiotic Armeniaspirols in Streptomyces armeniacus. J Nat Prod. https://doi.org/10.1021/acs.jnatprod.8b00753
